# Noninvasive Prediction of Programmed Cell Death Protein-Ligand 1 Expression in Locally Advanced Non-small Cell Lung Cancer by ^18^F-Fluorodeoxyglucose Positron Emission Tomography/Computed Tomography-Based Metabolic Habitats: A Multicenter Radiomic and Biological Study

**DOI:** 10.1245/s10434-025-18139-2

**Published:** 2025-08-29

**Authors:** Yu Ji, Kai Cui, Juntao Zhang, Jiaqi Wang, Zhengjun Dai, Yong Cui, Haojie Ge, Jingsong Zheng, Dexin Yu

**Affiliations:** 1 Department of Radiology, The Second Qilu Hospital of Shandong University, Jinan, Shandong China; 2 Department of Radiology, Qilu Hospital of Shandong University, Jinan, Shandong China; 3 Department of GMS Medical Affairs, GE Healthcare PDX, Shanghai, China; 4 Department of Cancer Medical Center, People’s Hospital of Chengwu County, Heze, Shandong China; 5 Department of Scientific Research, Huiying Medical Technology Co., Ltd, Beijing, China; 6 Department of AI Innovation, Aspire Information Technologies (Beijing) Limited, Beijing, China; 7 Department of PET/CT, Shandong Cancer Hospital and Institute, Shandong First Medical University and Shandong Academy of Medical Sciences, Jinan, Shandong China

**Keywords:** Locally advanced non-small cell lung cancer, PD-L1 expression, ^18^F-FDG PET/CT, Metabolic habitat model

## Abstract

**Background:**

Programmed cell death protein-ligand 1 (PD-L1) expression is an important marker for immunotherapy in locally advanced non-small cell lung cancer (LA-NSCLC). PD-L1 expression has a bi-directional positive feedback relationship with glycolysis status.

**Objective:**

This study aimed to develop a metabolic habitat model based on ^18^F-fluorodeoxyglucose positron emission tomography/computed tomography (^18^F-FDG PET/CT) images to predict PD-L1 expression levels in patients with LA-NSCLC, and to explore relevant biological characteristics.

**Methods:**

We included 219 patients from two independent centers and divided them into the training (*n* = 175) and testing (*n* = 44) cohorts. Tumors were segmented into four spatially distinct, biologically similar metabolic habitat subregions using the Otsu method. Radiomic characteristics and metabolic parameters were extracted from each habitat and used to generate multiple predictive models based on the Extra Trees classifier. Data from 1043 patients in The Cancer Genome Atlas database were used to analyze the genes associated with PD-L1 expression in NSCLC.

**Results:**

The metabolic habitat model exhibited the highest performance, with area under the curve values of 0.833 and 0.786 in the training and testing cohorts, respectively, outperforming other models. Subregion analysis revealed that high-glycolytic/high-density habitats (PET_High_–CT_High_) exhibited the highest metabolic characteristics, and their spatial distribution correlated positively with PD-L1 expression. Four genes (*IFNG*, *IL2RA*, *HK3*, and *MYCN*) were associated with PD-L1 expression in glycolysis gene correlation analysis.

**Conclusions:**

The metabolic habitat model based on ^18^F-FDG PET/CT enables noninvasive prediction of PD-L1 expression in LA-NSCLC. Its interpretability is enhanced by spatial habitat distribution, thereby advancing its potential for clinical translation.

**Supplementary Information:**

The online version contains supplementary material available at 10.1245/s10434-025-18139-2.

Locally advanced non-small cell lung cancer (LA-NSCLC) represents stage III NSCLC, which accounts for approximately 20–25% of NSCLCs.^1,2^ In the pre-immunotherapy era, despite aggressive radiotherapy and chemotherapy approaches, the progression of LA-NSCLC remained relatively slow and the prognosis was poor, with a 5-year survival rate of only 10–30%.^2,3^ Over the past several years, significant advances in immune checkpoint blockade have resulted in a paradigm shift for patients with lung cancer. Patients with both resectable and unresectable LA-NSCLC have benefited substantially from treatment with immune checkpoint inhibitors (ICIs).^1–5^ At present, the selection of immunotherapy needs to be based on practice target programmed death-ligand 1 (PD-L1) expression (tumor proportion score [TPS]), which is the only predictive biomarker available in clinical practice to date.^6–9^ However, this method has many limitations, such as sampling bias and poor repeatability due to the heterogeneous nature of tumors, the requirement of invasive biopsies, assays that are not rapid, considerable cost, and failure to yield actionable results due to insufficient quantity or quality of the tissue. Therefore, high-throughput, noninvasive longitudinal methods are urgently needed to predict programmed death-ligand 1 (PD-L1) expression.

^18^F-fluorodeoxyglucose positron emission tomography/computed tomography (^18^F-FDG PET/CT) is a noninvasive molecular imaging method widely used in the clinical diagnosis and treatment of LA-NSCLC.^6,10,11^ Previous studies have shown that glycometabolic rearrangements (aerobic glycolysis) and immune evasion are two hallmarks of cancer.^12,13^ Moreover, PD-L1 expression has a bidirectional positive feedback relationship with glycolysis status.^13,14^ Thus, ^18^F-FDG PET/CT, which can reveal glycolysis status, holds significant promise for predicting PD-L1 expression. In preliminary studies, simple metabolic parameters of ^18^F-FDG PET/CT, such as the maximum standardized uptake value (SUV_max_), mean SUV (SUV_mean_), metabolic tumor volume (MTV), and total lesion glycolysis (TLG), have shown predictive value for PD-L1 expression in NSCLC;^5,16^ however, these simple metabolic features do not fully capture the complexity and heterogeneity of NSCLC.^17^ Previous studies have demonstrated that ^18^F-FDG PET/CT-based radiomics has a high application value in predicting PD-L1 expression in NSCLC;^18,19^ however, these studies extracted radiomics features based on the whole tumor, assuming homogeneous tumor patterns, inevitably ignoring the density and metabolic differences caused by tumor heterogeneity, resulting in loss of image information and poor interpretability.^20^

Habitat analysis aims to capture spatial heterogeneity within tumors. Metabolic habitat images constructed based on ^18^F-FDG PET/CT can not only uncover subregional structural features but also reflect the degree of metabolism in each habitat and metabolic differences between habitats. Several studies have used habitat images from ^18^F-FDG PET/CT to identify benign and malignant lesions,^21^ classify pathologic subtypes,^22^ evaluate immunotherapy,^23^ and predict recurrence^17^ in NSCLC. These studies have demonstrated that metabolic habitats can more effectively capture microenvironmental changes, and their modeling efficacy is superior to that of conventional radiomics. However, to the best of our knowledge, no study has yet systematically elucidated the association between different metabolic habitat radiomics and PD-L1 expression in LA-NSCLC.

We hypothesized that metabolic habitats constructed from ^18^F-FDG PET/CT could reflect the morphological and functional characteristics of different subregions and might provide incremental value for noninvasive prediction of PD-L1 expression. In this work, we developed imaging biomarkers for predicting immune status by modeling the prediction of PD-L1 from habitat radiomic features obtained from different metabolic habitats of LA-NSCLC. In addition, the potential biological characteristics of NSCLC were initially explored through interpretive analysis of each metabolic habitat and differential glycometabolism-related genes.

## Methods

### Study Cohorts

**Fig. 1:**
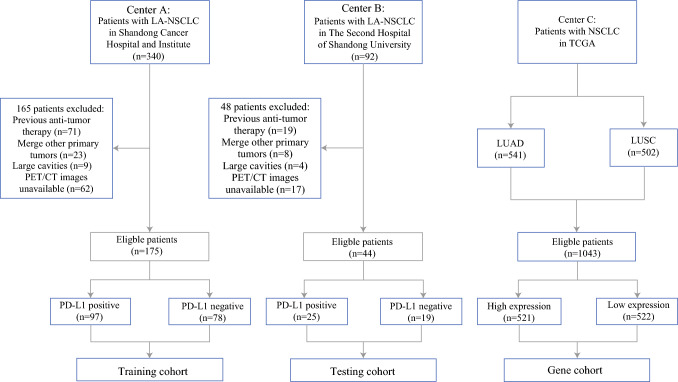
Patient enrollment and study profile. *LA-NSCLC* locally advanced non-small cell lung cancer, *TCGA* The Cancer Genome Atlas, *PET* positron emission tomography, *CT* computed tomography, *PD-L1* programmed death-ligand 1, *LUAD* lung adenocarcinoma, *LUSC* lung squamous cell carcinoma

With Institutional Review Board approval, we retrospectively collected data of patients with NSCLC treated consecutively at two medical centers from January 2017 to June 2024. The inclusion criteria were (1) biopsy-confirmed NSCLC; (2) clinical stage III; (3) baseline ^18^F-FDG PET/CT scan before treatment; and (4) PD-L1 expression detection. Exclusion criteria included (1) previous induction chemotherapy or surgery; (2) other primary tumors; (3) incomplete or poor-quality ^18^F-FDG PET/CT imaging; and (4) large cavities within tumors. Detailed information of the cohorts in this study is provided in electronic supplementary material (ESM) Methods. The flowchart details the process of patient inclusion in this retrospective study (Fig. 1). TNM staging of LA-NSCLC in this study was based on the International Association for the Study of Lung Cancer (IASLC) Lung Cancer Staging Project.^24^

### Programmed Death-Ligand 1 (PD-L1) Detection by Immunohistochemistry

For both the training and testing cohorts, PD-L1 staining was conducted using the Dako Link 48 platform and Dako 22C3 antibody to quantify PD-L1. The level of PD-L1 expression was presented as a TPS, indicating the percentage of viable tumor cells showing membrane PD-L1 staining relative to all viable tumor cells. PD-L1 positivity was defined as a TPS ≥ 1%. The PD-L1 test results were acquired retrospectively. To minimize reader bias, all staining results were reviewed and analyzed by two experienced pathologists who were blinded to each other’s scores and unaware of the patients’ clinical information. In cases of discrepancies, pathologists discussed their findings to reach a consensus.

### ^18^F-Fluorodeoxyglucose Positron Emission Tomography/Computed Tomography Image Acquisition and Tumor Segmentation

Imaging was conducted using multiple PET/CT scanners (TF Big Bore, Philips, Holland; Ingenuity TF, Philips, Holland; and Biograph Horizon, Siemens, Germany). To address the heterogeneity arising from variations in imaging scanners/protocols and the inherently lower resolution of PET/CT images, we implemented a five-stage preprocessing pipeline: intensity normalization, spatial registration, resampling, super-resolution reconstruction, and image discretization. Full specifications of scanning protocols and preprocessing details are provided in the ESM Methods.

The identification and segmentation of target tumor regions were meticulously conducted by experienced clinical radiologists, leveraging their expertise and available clinical datasets. The tumor segmentation of all images was completed using 3D Slicer through a manual process. Detailed information on tumor segmentation is provided in the ESM Methods.

### Tumor Metabolic Habitat Generation

**Fig. 2:**
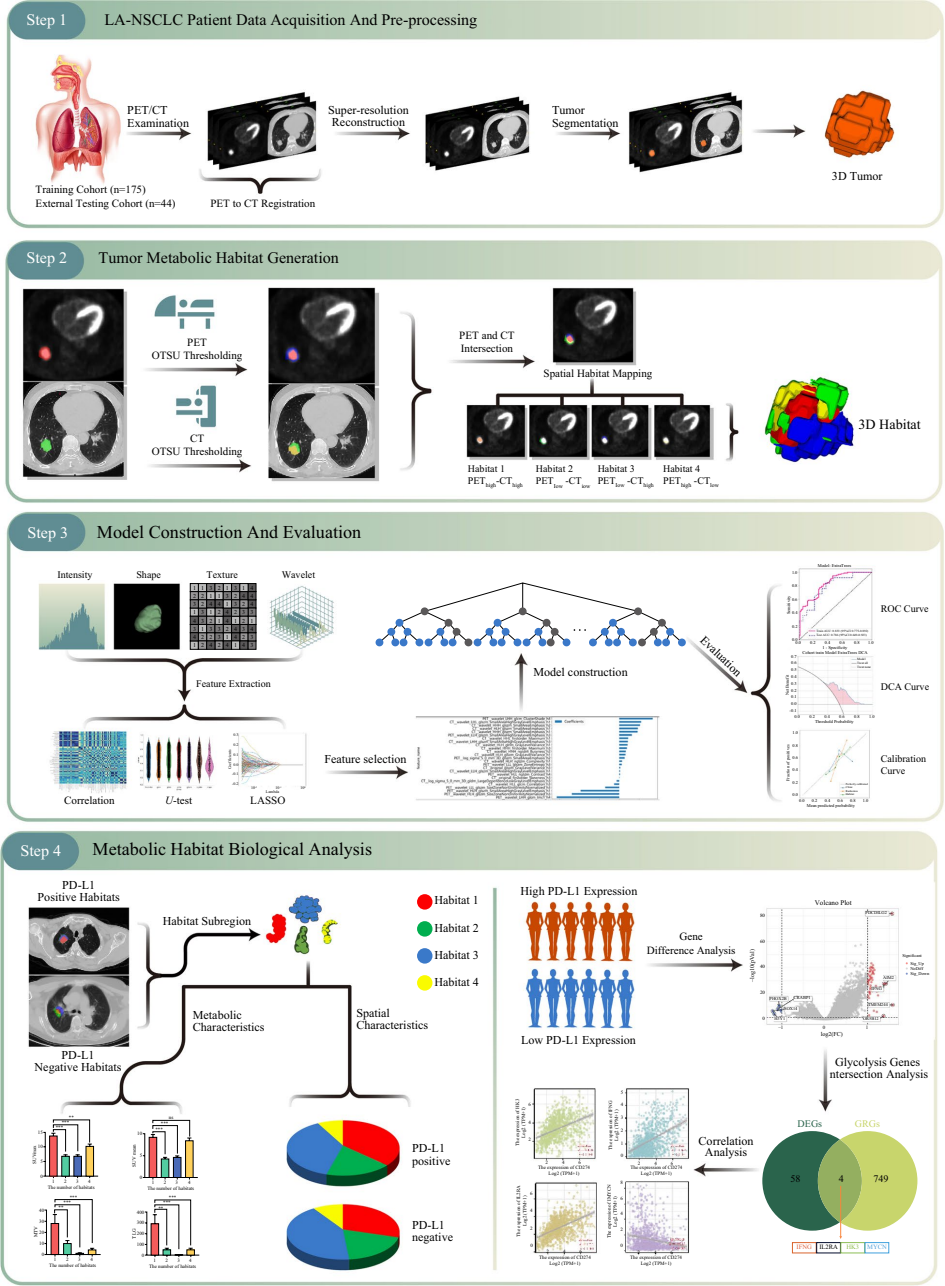
Study design of metabolic habitat imaging-based radiomic analysis of patients with LA-NSCLC. This retrospective study was conducted in four steps. *Step 1:* LA-NSCLC patient data acquisition. The preprocessing involved ^18^F-FDG PET/CT registration and fusion, image normalization and resampling, and super-resolution reconstruction. *Step 2:* Tumor metabolic habitat generation. Habitat segmentation was conducted using the Otsu binary classification algorithm and resulted in four distinct habitat subregions. *Step 3:* Model construction and evaluation. Image features from each metabolic habitat were extracted and a predictive model for PD-L1 expression was developed based on Extra Trees. *Step 4:* Metabolic habitat biological analyses. The metabolic characteristics and spatial distribution of each habitat subregion, as well as glycolytic genes, were analyzed to explore the biological characteristics of NSCLC. *LA-NSCLC* locally advanced non-small cell lung cancer, ^*18*^*F-FDG PET/CT* ^18^F-fluorodeoxyglucose positron emission tomography/computed tomography, *PD-L1* programmed death-ligand 1, *3D* three-dimensional, *LASSO* least absolute shrinkage and selection operator, *ROC* receiver operating characteristic, *DCA* decision curve analysis, *DEGs* differentially expressed genes, *GRGs* glycolysis-related genes

Habitat segmentation was conducted using the Otsu binary classification algorithm, an unsupervised method that performs segmentation based on the grayscale values of the volume of interest (VOI) regions without requiring prior knowledge. For the CT image VOI region, the Otsu algorithm calculates the maximum interclass variance of the grayscale values within the region to determine a threshold. It then segments the VOI into high- and low-grayscale regions based on this threshold. The same method was applied to the VOI regions of the PET images, segmenting them into high- and low-grayscale regions. Finally, the intersection of the segmented CT and PET habitats resulted in four distinct habitat subregions: high-glycolytic/high-density (PET_high_–CT_high_), low-glycolytic/low-density (PET_low_–CT_low_), low-glycolytic/high-density (PET_low_–CT _high_), and high-glycolytic/low-density (PET_high_–CT_low_) subregions. The specific display is shown in Fig. 2.

### Radiomics Feature Extraction and Selection

Radiomic features were extracted using an in-house feature analysis program embedded in the Pyradiomics package (http://pyradiomics.readthedocs). In accordance with the Imaging Biomarker Standardization Initiative (IBSI),^25^ 1015 image-based radiomic features were extracted from each region of interest, including each habitat and the entire tumor region. Detailed information on these features is provided in the ESM figures.

To reduce the dimensionality of the radiomics features and select the important features for the prediction model, a three-step feature selection procedure was performed. First, a two-sample Mann–Whitney U test was used to preselect the radiomics features that were significantly (*p* < 0.05) different between PD-L1-positive and PD-L1-negative expression. Next, the Spearman rank correlation coefficient was used to reduce redundancy. If the absolute value of the correlation coefficient between any two features was > 0.9, only one was retained. Finally, a least absolute shrinkage and selection operator (LASSO) procedure with tenfold cross-validation was used to select the most useful predictive features with non-zero coefficients.

### Model Development

Three prediction models were constructed based on tumor imaging and clinical features: the metabolic habitat, whole-tumor, and clinical models. Feature selection was performed based on each metabolic habitat and whole-tumor region. The selected features were then input into the Extra Trees (ET) machine learning model to construct metabolic habitat and whole-tumor prediction models for PD-L1 expression. We selected the ET model as a prototype because of its robustness, variance reduction, and efficiency.^26^ In addition, we incorporated the screened features into multiple machine learning models for verification and effectiveness evaluation. The machine learning models involved in this study were built using the scikit-learn package (version 1.0.2) in Python (version 3.7.9). Univariate logistic regression was performed on conventional PET/CT characteristics of tumors and patient clinical characteristics to assess their correlation with PD-L1 expression. Characteristics with a *p* value < 0.05 were included to develop clinical predictive models. The effectiveness of these models was evaluated and compared using receiver operating characteristic (ROC) curve analysis.

### Glucose Metabolism Gene Analysis

Transcriptome data from 541 patients with lung adenocarcinoma (LUAD) and 502 patients with lung squamous cell carcinoma (LUSC) were obtained from The Cancer Genome Atlas (TCGA) database (http://cancergenome.nih.gov/) for exploratory analysis of genes related to PD-L1 expression in NSCLC. Based on previous studies of glycolysis-related genes,^27^ we included 753 glycolysis-related genes for subsequent analysis. First, we removed the batch effect between the LUAD and LUSC datasets using the ComBat function within the sva package. Next, differential gene expression analysis between the PD-L1 high- and low-expression groups was performed using the ‘limma’ package, and *p* values were adjusted for multiple testing using the Benjamini–Hochberg method. Genes with an FDR < 0.05 were considered statistically significant. These differentially expressed genes were then intersected with glycolysis-related genes to identify differentially expressed glycolysis-related genes. Finally, we performed Spearman correlation analysis to investigate the relationship between PD-L1 expression and these glycolysis-related genes.

### Statistical Analysis

The R software package (v3.5.3; https://www.r-project.org/) was used for statistical analysis. For continuous variables, means and standard deviations or medians with interquartile ranges were calculated. For categorical variables, absolute numbers with percentages were recorded. The independent samples t-test or Mann–Whitney U test were used to compare quantitative data. Pearson’s Chi-square or Fisher’s exact tests, where appropriate, were used to compare the difference in qualitative data. All tests were two-sided, and a *p* value < 0.05 was considered statistically significant; confidence intervals (CIs) for proportions are reported as two-sided exact binomial 95% CIs.

## Results

### Patient Characteristics According to the Multicenter Data

The demographic and clinical characteristics of the patients in the training and testing cohorts are shown in Table 1. A statistically significant difference was observed in the T stage of LA-NSCLC in both the training and testing cohorts, whereas the remaining features did not differ significantly. The results of the univariate and multivariate analyses of the clinical and metabolic parameters used to predict PD-L1 expression are summarized in ESM Table 1. Among the clinical and metabolic parameter predictors, tumor histology (odds ratio [OR] 0.535, 95% CI 0.310–0.921; *p* < 0.05) and neuron-specific enolase (NSE; OR 1.735, 95% CI 1.003–3.001; *p* < 0.05) were significantly associated with PD-L1 positivity (ESM Table 1).

**Table 1:** Demographic and clinical characteristics of the study cohort

Parameter	Training cohort [*n* = 175]	Testing cohort [*n* = 44]	*p* Value
Age, years			0.348
Mean ± SD	62.9 ± 10.0	64.4 ± 8.1	
Range	35–85	35–85	
Sex			0.383
Male	138 (78.9)	32 (72.7)	
Female	37 (21.1)	12 (27.3)	
Smoking history			0.388
Yes	107 (61.1)	30 (68.2)	
No	68 (38.9)	14 (31.8)	
Family history			0.936
Yes	11 (6.3)	2 (4.5)	
No	164 (93.7)	42 (95.5)	
Tumor histology			0.975
LUAD	80 (45.7)	20 (45.5)	
LUSC	95 (54.3)	24 (54.5)	
Tumor location			0.219
Central	113 (64.6)	24 (54.5)	
Peripheral	62 (35.4)	20 (45.5)	
T staging			
I	16 (9.1)	9 (20.4)	0.035
II	72 (41.1)	17 (38.6)	0.762
III	47 (26.9)	9 (20.5)	0.384
IV	40 (22.9)	9 (20.5)	0.732
N staging			
0	3 (1.7)	2 (4.5)	0.302
1	22 (12.6)	3 (6.8)	0.283
2	91 (52.0)	27 (61.4)	0.265
3	59 (33.7)	12 (27.3)	0.414
Clinical staging			
IIIA	79 (45.1)	25 (56.8)	0.166
IIIB	71 (40.6)	13 (29.5)	0.179
IIIC	25 (14.3)	6 (13.6)	0.912

Data are expressed as number of participants (%) unless otherwise specified

*LUAD* lung adenocarcinoma, *LUSC* lung squamous cell carcinoma, *SD* standard deviation

### Performance Evaluation of Different Predictive Models

**Fig. 3:**
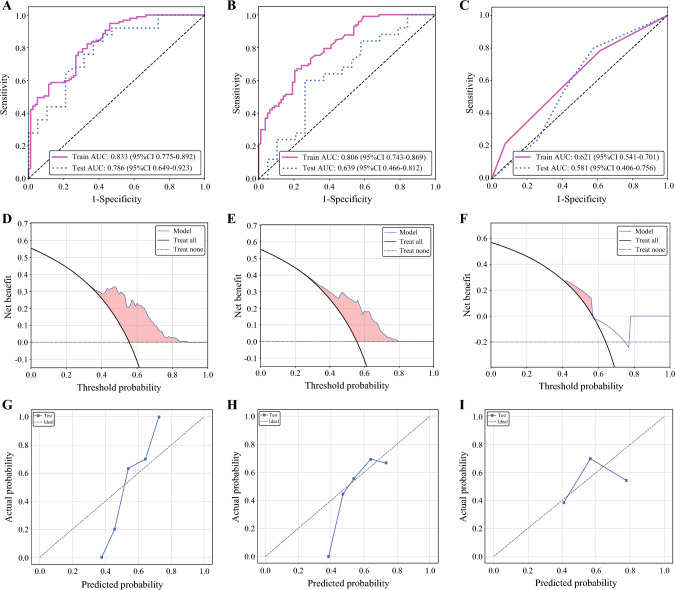
Performance comparison of the metabolic habitat, whole-tumor, and clinical models. The metabolic habitat model (**a**) performed the best, followed by the whole-tumor model (**b**), while the clinical model (**c**) performed the poorest. Decision curve analyses (**d–f**) and calibration curves (**g–i**) for the three models. In a reasonable threshold probability range, the metabolic habitat model typically generates a greater overall net benefit than the other models. *AUC* area under the receiver operating characteristic curve, *CI* confidence interval

To distinguish PD-L1-positive from PD-L1-negative expression, the metabolic habitat model demonstrated robust performance, with an area under the ROC curve (AUC) of 0.833 (95% CI 0.775–0.892) in the training cohort and 0.786 (95% CI 0.649–0.923) in the testing cohort. The model achieved accuracies of 74.9% (95% CI 67.9–81.0%) and 72.7% (95% CI 58.1–84.0%), sensitivities of 81.4% (72.2–88.7%) and 80.0% (61.5–90.9%), and specificities of 66.7% (95% CI 56.1–76.3%) and 63.2% (95% CI 40.9–82.3%) for the training and testing cohorts, respectively. We compared the predictive performance of the metabolic habitat model with that of the whole-tumor and clinical models using AUC, calibration, and decision curves. The results revealed that the metabolic habitat model exhibited the highest performance, followed by the whole-tumor model, with the clinical model exhibiting the lowest performance (Fig. 3). In the training cohort, the AUC values were ranked as follows: 0.833 > 0.806 > 0.621, whereas in the testing cohort, the sequence was 0.786 > 0.639 > 0.581 (ESM Table 2).

### Identification of Habitat Subregions with Different Metabolic Characteristics

**Fig. 4:**
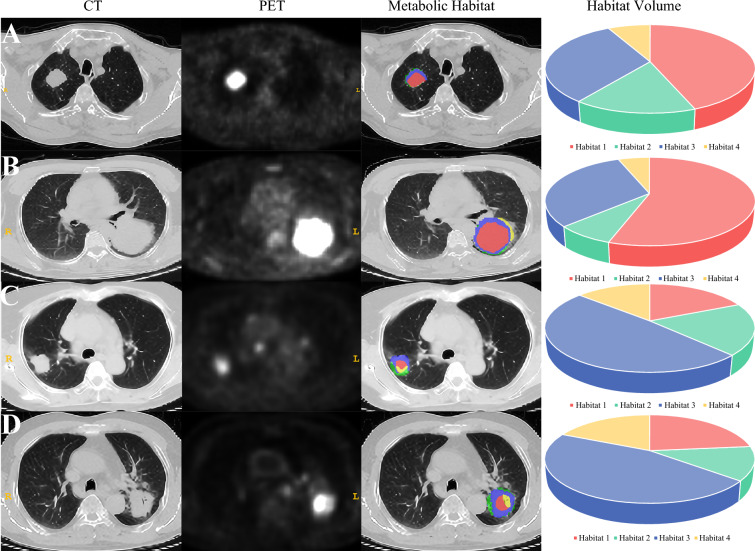
Representative ^18^F-FDG PET/CT and metabolic habitat images of two PD-L1-positive expression patients (**a, b**) and two PD-L1-negative expression patients (**c, d**). Note: (1) We mapped each metabolic habitat onto CT images, annotating each habitat subregion. (2) Habitat 1 (red) represented high-glycolytic/high-density habitats; Habitat 2 (green) represented low-glycolytic/low-density habitats; Habitat 3 (blue) represented low-glycolytic/high-density habitats; and Habitat 4 (yellow) represented high-glycolytic/low-density habitats. ^*18*^*F-FDG PET/CT* ^18^F-fluorodeoxyglucose positron emission tomography/computed tomography, *PD-L1* programmed death-ligand 1

**Fig. 5:**
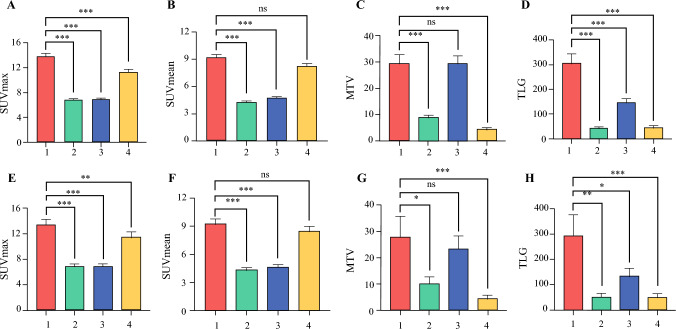
Comparison of metabolic parameters in different habitats. In the training (**a–d**) and testing (**e–h**) cohorts, Habitat 1 showed the highest SUV_max_ (**a**, **e**) and TLG (**d**, **h**), which exceeded those of the other habitats. Habitats 1 and 4 had the highest SUV_mean_, exceeding those of Habitats 2 and 3 (**b**, **f**). MTV values were significantly elevated in Habitats 1 and 3 relative to Habitats 2 and 4 (**c**, **g**). Habitat 1 (red) represented high-glycolytic/high-density habitats; Habitat 2 (green) represented low-glycolytic/low-density habitats; Habitat 3 (blue) represented low-glycolytic/high-density habitats; and Habitat 4 (yellow) represented high-glycolytic/low-density habitats. *SUV*_*max*_ maximum standardized uptake value, *TLG* total lesion glycolysis, *SUV*_*mean*_ mean standardized uptake value, *MTV* metabolic tumor volume, *ns* non-significant

We identified four distinct metabolic habitats within the tumor, maintaining consistency across both the training and testing cohorts, as shown in Fig. 2. Each habitat was mapped separately onto PET and CT images, with specific density and metabolic characteristics annotated for each subregion (Fig. 4). For the high-glycolytic/high-density habitat, PET SUV was high, and the number of CT Hounsfield units (HUs) was also high; for low-glycolytic/low-density habitats, the PET SUV and CT HU numbers were low; for low-glycolytic/high-density habitats, the PET SUV was low, whereas the CT HU number was high; and for high-glycolytic/low-density habitats, the PET SUV was high, whereas the CT HU number was low. The representative characteristics and statistical analyses of each habitat are presented in Fig. 5. Across both cohorts, Habitat 1 consistently exhibited higher SUV_max_ and TLG values compared with all other habitats (*p* < 0.05). Habitats 1 and 4 showed comparable SUV_mean_, both significantly exceeding Habitats 2 and 3 (*p* < 0.001). MTV values were significantly elevated in Habitats 1 and 3 relative to Habitats 2 and 4 (*p* < 0.001).

### Correlation between Metabolic Habitat Spatial Characteristics and PD-L1 Expression

We analyzed the correlation between the spatial characteristics (voxel values and volume fraction) of each metabolic habitat and PD-L1 expression, using 1000 voxels as a unit. Univariate logistic regression analysis revealed that both the number of voxels (OR 1.014, 95% CI 1.001–1.027; *p* < 0.05) and the volume fraction (OR 8.84, 95% CI 1.069–73.125; *p* < 0.05) of high-glycolytic/high-density habitats were positively correlated with PD-L1 expression. In contrast, no significant correlation was found between PD-L1 expression and the other metabolic habitats (Table 2).

**Table 2:** Correlation of the spatial characteristics of each metabolic habitat with PD-L1 expression

Tumor habitats	OR	95% CI	*p* Value
Numbers of voxels (× 10^3^)			
Habitat 1	1.014	1.001–1.027	0.042
Habitat 2	1.002	0.968–1.038	0.903
Habitat 3	1.003	0.992–1.015	0.586
Habitat 4	0.977	0.946–1.008	0.143
Volume fraction			
Habitat 1	8.84	1.069–73.125	0.043
Habitat 2	1.481	0.231–9.513	0.679
Habitat 3	0.328	0.054–2.003	0.227
Habitat 4	0.277	0.031–2.456	0.249

ORs reported here indicate the relative change in odds that a 1-unit (1000 voxels) increase in each imaging parameter incurs

Habitat 1 = high-glycolytic/high-density habitat; Habitat 2 = low-glycolytic/low-density habitat; Habitat 3 = low-glycolytic/high-density habitat; and Habitat 4 = high-glycolytic/low-density habitat

*PD-L1* programmed death-ligand 1, *OR* odds ratio, *CI* confidence interval

### Identification and Analysis of Differentially Expressed Glycolysis-Related Genes

**Fig. 6:**
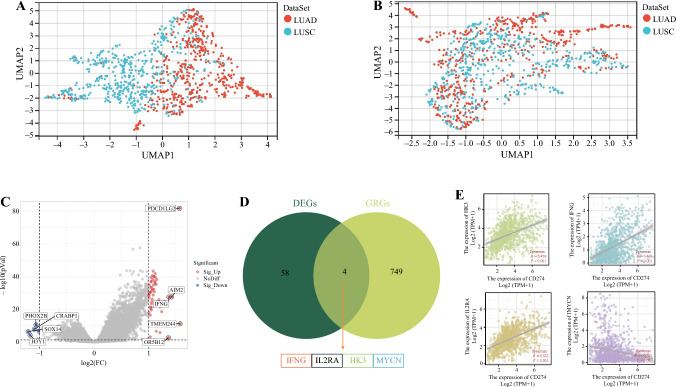
Correlation analysis of differentially expressed PD-L1 genes in NSCLC. UMAP maps of NSCLC PD-L1 high- and low-expression groups before (**a**) and after (**b**) batch removal from TCGA. Volcano map of differentially expressed genes (**c**). The Venn diagram shows that four glycolysis-related genes (*IFNG, IL2RA, HK3,* and *MYCN*) were associated with the differential expression of PD-L1 in NSCLC according to the intersection of the differentially expressed genes and glycolysis-related genes (**d**). The relationship between four glycolytic genes and PD-L1 expression (**e**). *PD-L1* programmed death-ligand 1, *NSCLC* non-small cell lung cancer, *TCGA* The Cancer Genome Atlas, *LUAD* lung adenocarcinoma, *LUSC* lung squamous cell carcinoma, *DEGs* differentially expressed genes, *GRGs* glycolysis-related genes

We first integrated the LUAD and LUSC transcriptome data from TCGA. The UMAP plot (Fig. 6a, b) shows that the samples of the two datasets before the removal of the batch effect were clustered together individually, indicating a batch effect, whereas the samples were clustered together and intertwined after the removal of the batch effect, suggesting effective batch effect removal. Next, we identified differentially expressed genes in PD-L1-high- and PD-L1-low-expressing tumors, revealing 53 upregulated and 9 downregulated genes (Fig. 6c). Finally, to explore the role and potential pathways of glycolysis in NSCLC, we intersected these differentially expressed genes with glycolytic genes and identified four glycolysis-related genes (*IFNG, IL2RA, HK3, MYCN*) associated with PD-L1 differential expression (Fig. 6d). On this basis, we further analyzed the correlations between PD-L1 expression and the expression of these glycolysis-related genes. PD-L1 expression was positively correlated with IL2RA (*R* = 0.522, *p* < 0.001), IFNG (*R* = 0.468, *p* < 0.001), and HK3 (*R* = 0.458, *p* < 0.001), and negatively correlated with MYCN (*R* = −0.201, *p* < 0.001) [Fig. 6e].

## Discussion

In this study, we developed a metabolic habitat model that integrated the features of PET and CT images to characterize tumor subregions (habitats) with metabolic heterogeneity. By extracting microscopic radiomics features of different subregions, we were able to predict the PD-L1 expression status of patients with LA-NSCLC. The results of this study demonstrated that the metabolic habitat-based prediction model exhibited high efficacy, with an AUC of 0.833 for the training cohort and 0.786 for the validation cohort. This model outperformed both the whole-tumor and clinical models, indicating its potential as a noninvasive imaging marker for PD-L1 detection. Additionally, this study provided biological validation based on metabolic habitats, supporting the hypothesis that high glycolytic characteristics serve as cancer markers mediating immune escape in LA-NSCLC. Furthermore, we analyzed glycolytic genes related to PD-L1 expression, revealing longitudinal crosstalk between genes, metabolism, and molecular imaging, and providing a theoretical foundation for subsequent metabolic immunotherapy.

As core features of NSCLC, the rearrangement of glucose metabolism and immune escape (PD-L1 expression) have a mutually positive regulatory relationship, significantly influencing the biological behavior of tumors.^13,14,28^ Parameters of FDG PET/CT, a non-invasive routine assessment method, can effectively reflect the glycometabolic status and morphological features of LA-NSCLC, making them ideal PD-L1 predictive markers. Previous studies have used metabolic parameters of ^18^F-FDG PET/CT to predict PD-L1 expression,^15,16,29,30^ however these studies have not reached a unified conclusion. Our study also did not find evidence that metabolic parameters can predict PD-L1 expression. Theoretically, high PD-L1 expression in tumor cells upregulates the glycolysis pathway,^13^ and FDG PET-based metabolic parameters (especially the SUV_max_) can be used to predict PD-L1 status. However, in related studies where metabolic parameters predict immune expression, their efficacy has not been satisfactory. This may be due to the multifaceted causes of FDG uptake in NSCLC, not solely the expression of PD-L1, and complex microenvironmental and metabolic mechanisms likely dominate.

In recent years, numerous researchers have investigated the utility of ^18^F-FDG PET/CT radiomics as a noninvasive approach for predicting PD-L1 expression status, achieving promising diagnostic performance with AUC values ranging from 0.604 to 0.970.^19,31–33^ This radiomics-based assessment represents a significant advancement over previous studies of metabolic parameters: on the one hand, the comprehensive utilization of a large number of microscopic features in medical images, and on the other hand, the complementarity with clinical features and conventional metabolic parameters. Nevertheless, conventional radiomics extracts features from the entire tumor region, based on the theory of uniform distribution of tumor heterogeneity, exhibiting substantial performance variability and suffering from limited interpretability. To address these limitations, we developed an ^18^F-FDG PET/CT metabolic habitat-based radiomics model by dividing LA-NSCLC into subregions (metabolic habitats) with similar biological features. By quantifying the radiomics features and metabolic information within each metabolic habitat, we established predictable connections among macroscopic tumor features and microscopic tumor cells, molecules, and the microenvironment, which achieved a comprehensive assessment of tumor biological information. Compared with conventional radiomics studies that consider the tumor as a whole, this approach can adequately evaluate the spatial metabolic heterogeneity within tumors. This not only improves the stability and efficacy of radiomics models but also increases their interpretability.

This study used a combination of metabolic functional imaging (PET), morphological imaging (CT) and habitat technology (subregion analysis) to characterize PD-L1 in LA-NSCLC, which is one of the main highlights of this study. For habitat segmentation of the tumor region, we used the Otsu algorithm, which has better interpretability and repeatability than other algorithms.^34^ In addition, to fully utilize the information of medical images, we used super-resolution reconstruction technology to improve the quality of the image, which has been proven to improve model performance.^35,36^ Jiang et al. used radiomics to assess PD-L1 expression levels in 399 cases of NSCLC and concluded that radiomic features of PET images did not improve the efficacy of the model.^33^ These authors attributed this result to the low resolution of the PET/CT images, a conclusion similar to that reached by Mu et al.^37^ Therefore, we consider super-resolution reconstruction technology to be essential for processing PET/CT images, as it can prevent the loss of PET image information and improve model efficiency. The results of our study confirmed this (ESM Fig. 8). Overall, the integration of metabolic and morphologic imaging, habitat segmentation, and advanced image reconstruction technologies offers a comprehensive approach for characterizing PD-L1 expression in LA-NSCLC, providing a robust and interpretable predictive model.

Our analysis of individual subregions with similar or identical biological significance can better explain the biological behavior of LA-NSCLC, which is another highlight of this study. We analyzed the metabolic parameters and spatial characteristics of each habitat. Habitat 1 (high-glycolytic/high-density) exhibited elevated PET and CT signals, indicating densely cellular regions with active glycolysis. This habitat demonstrated the highest SUV_max_ and TLG, signifying heightened energy demands potentially linked to tumor proliferation or immune evasion. These features may enhance their utility for PD-L1 expression prediction, aligning with prior clinical studies.^38,39^ In contrast, Habitat 2 (low-glycolytic/low-density) and Habitat 3 (low-glycolytic/high-density) showed reduced metabolic activity, suggesting limited tumor proliferation or necrotic components. Such microenvironments may correlate with lower PD-L1 expression probability. Habitat 4 (high-glycolytic/low-density), typically localized at tumor peripheries, displayed high glycolysis but low cellular density. This profile likely reflects complex microenvironments involving inflammatory infiltration and stromal interactions.^40^ Despite metabolic activity, heterogeneity and low TLG diminish its reliability for PD-L1 characterization. Based on these findings, we concluded that metabolic parameters may be more meaningful when stratified by metabolic level. Moreover, the larger the volume fraction of high-glycolytic/high-density habitat within the tumor, the more likely it is to express PD-L1. This finding revealed that habitats’ metabolic parameters and spatial distribution are closely related to PD-L1 expression and that their precise quantification can better predict PD-L1 status. Building on these findings, metabolic habitat mapping offers a practical solution to mitigate spatial sampling bias in PD-L1 assessment. During biopsy procedures, targeting high-glycolytic/high-density habitats while avoiding metabolically reduced areas optimizes sampling accuracy. Furthermore, multi-regional sampling better captures tumor heterogeneity in LA-NSCLC, significantly reducing false-negative rates. This approach aligns with clinical needs for precision biopsy while leveraging routine PET/CT data without additional costs.

In this study, we explored glucose metabolism-related genes upstream of metabolic heterogeneity in two steps, resulting in four related glucose metabolism genes: *HK3, IFNG, IL2RA* and *MYCN*. Previous studies have shown that all these glycometabolism genes are associated with immune escape in tumors,^41–43^ and their differential expression also causes differences in metabolic parameters on PET/CT images.^44^ *HK3*, *IFNG*, and *IL2RA* are associated with tumor proliferation, metastasis, presentation of indicated antigens, protein expression, and T-cell regulation and can promote PD-L1 expression; among them, *IL2RA* can regulate the classical Akt/mTOR pathway.^43^ In this study, *MYCN* was found to be negatively correlated with PD-L1 expression, which may be related to the activation of multiple contradictory energy metabolic pathways (aerobic glycolysis, tricarboxylic acid cycle, oxidative phosphorylation, and glutaminolysis).^45^ This study preliminarily explored the immunoregulatory role of genes related to glucose metabolism, which can also be used to assist immunotherapy by modulating glucose metabolism pathways in the future.

The metabolic habitat model constructed based on ^18^F-FDG PET/CT in this study has two advantages. Clinically, this model can serve as a noninvasive biomarker of PD-L1 status, which is superior to other noninvasive methods (traditional radiomics and clinical features). For patients with high predictive confidence, unnecessary biopsies may be reduced, thereby lowering procedural risks and potential complications. Additionally, it offers a viable alternative for patients ineligible for invasive procedures, enabling immunotherapy decisions based on noninvasive imaging. Notably, the PD-L1-associated metabolic habitat (high-glycolytic/high-density subregion) provides actionable guidance for biopsy targeting, minimizing sampling errors caused by tumor heterogeneity. Methodologically, it can be used as an upgraded application of conventional radiomics to quantify the morphological and functional differences caused by intratumoral precisely. Additionally, it explains the biological significance of tumors.

Nevertheless, this study has several limitations. First, its retrospective design inevitably involves the possibility of clinical data loss or selection bias, necessitating large-scale prospective studies for model validation. Second, while the testing cohort (*n* = 44) successfully validated our model’s performance (AUC 0.786), its limited size necessitates caution in extrapolating results to broader populations. Small testing cohorts may overestimate predictive efficacy due to reduced statistical power and increased susceptibility to sampling bias, therefore necessitating future multi-institutional studies with larger cohorts to confirm generalizability. Third, some of the PD-L1 expression detected in this study was derived from puncture pathology. Although these findings align with those of clinical practice, and previous studies have shown high consistency between puncture and surgical samples,^46,47^ spatial heterogeneity in NSCLC may introduce sampling bias, particularly for tumors with low PD-L1 expression.^48^ Future studies incorporating multiregional biopsy or liquid biopsy-based PD-L1 profiling are warranted to mitigate this limitation. Finally, the metabolic habitat demonstrated biological value in this study, and further well-established pathological, genetic, and mechanistic studies are needed to confirm its accuracy. Future studies should investigate the relationship between metabolic habitats and immunotherapy response/prognosis to address tumor microenvironment heterogeneity.

## Conclusion

In this study, we developed and validated a metabolic habitat model that can assess the PD-L1 expression status of LA-NSCLC by characterizing the spatial metabolic heterogeneity of tumors based on ^18^F-FDG PET/CT images. This model has demonstrated good predictive efficacy, along with the advantages of being convenient and noninvasive, providing a more comprehensive reflection of the biological behavior of tumors. It can serve as an effective alternative method for LA-NSCLC immunohistochemical detection. In addition, our habitat model integrates with standard PD-L1 testing to refine patient immunotherapy stratification and guiding targeted biopsies. This approach augments precision oncology workflows by transforming routine PET/CT scans into actionable maps for treatment selection algorithms.

## Supplementary Information

Below is the link to the electronic supplementary material.

Supplementary file1 (DOC 10498 KB)

## Data Availability

All data from TCGA can be downloaded from TCGA website. Other data are available from the corresponding author upon reasonable request.
