# Supplementary material for: Noninvasive Prediction of Programmed Cell Death Protein-Ligand 1 Expression in Locally Advanced Non-small Cell Lung Cancer by 18F-Fluorodeoxyglucose Positron Emission Tomography/Computed Tomography-Based Metabolic Habitats: A Multicenter Radiomic and Biological Study
Source: Ann Surg Oncol. 2025 Aug 29;32(13):10094–107. doi: 10.1245/s10434-025-18139-2 (PMC12589310; doi:10.1245/s10434-025-18139-2)
Supplement: Supplementary file 1 — Supplementary file1 (DOC 10498 KB) [file 10434_2025_18139_MOESM1_ESM.doc]

**Supplementary Information**

**Title:**

Noninvasive Prediction of PD-L1 Expression in LA-NSCLC by 18F-FDG PET/CT-Based Metabolic Habitats: A Multicenter Radiomic and Biological Study

**Authors:**

Yu Ji^1,2^, Kai Cui^1^, Juntao Zhang^3^, Jiaqi Wang^4^ Zhengjun Dai^5^, Yong Cui^1^, Haojie Ge^6^, Jingsong Zheng^7^, Dexin Yu^2*^

1. Department of Radiology, The Second Hospital, Cheeloo College of Medicine, Shandong University, Jinan, Shandong, China.

2. Department of Radiology, Qilu Hospital of Shandong University, Jinan, Shandong, China.

3. Department of GMS medical affairs, GE Healthcare PDX, Shanghai, China.

4. Department of Cancer Medical Center, People's Hospital of Chengwu County, Heze, Shandong.

5 Department of Scientific Research, Huiying Medical Technology Co., Ltd, Beijing

6. Department of AI Innovation, Aspire Information Technologies (Beijing) Limited, Beijing

7. Department of PET/CT, Shandong Cancer Hospital and Institute, Shandong First Medical University and Shandong Academy of Medical Sciences, Jinan, Shandong, China.

7. Department of Radiology, Qilu Hospital of Shandong University, Jinan, Shandong, China.

***Corresponding author:**

Dexin Yu

ORCID: 0000-0002-3430-4817

Department of Radiology,

Qilu Hospital of Shandong University, Jinan, Shandong, 250012, China

E-mail: [yudexin0330@sina.com](mailto:yudexin0330@sina.com)

**Content:**

Supplemental methods

Supplementary Figures 1-8

Supplementary Tables 1-4

**Supplemental Methods**

**Study cohorts**

The patients were screened according to these criteria for LA-NSCLC, ultimately 219 patients were included in the study, and divided into the training cohort and testing cohort according to medical institution. The first cohort (training cohort) comprised 175 cases of LA-NSCLC from Shandong Cancer Hospital and Institute, with 97 cases positive and 78 cases negative for PD-L1 expression. The second cohort (external testing cohort) consisted of 44 cases of LA-NSCLC from the Second Hospital of Shandong University, with 25 cases positive and 19 cases negative for PD-L1 expression. The third cohort (gene cohort) included 1043 cases of NSCLC from the TCGA, comprising 541 cases of LUAD and 502 cases of LUSC, which was used for preliminary exploration of immune-related glucose metabolism genes and potentially biological characteristics.

**^18^F-FDG PET/CT image acquisition and pre-processing**

^18^F-FDG with a pH of 5–7 and a radiochemical purity exceeding 95% was produced using a cyclotron (MINItrace, GE Healthcare, Milwaukee WI, USA). Patients fasteed for at least 6h and had blood glucose levels below 200 mg/dL prior to injection with 18F-FDG. Patients lay in a quiet room 60 min after intravenous injection with 4.4–5.5 MBq/kg 18F-FDG. Spiral CT scanning was performed at 120 kVp and 300 mA·s, followed by PET scans without repositioning. PET images were obtained at 7 to 8 couch positions per patient, with an acquisition time of 2 min per position. CT data were used for attenuation correction of PET images, and the attenuation-corrected PET and CT images were fused.

Given the variations of imaging scanners and protocols, as well as the lower resolution of PET/CT images, we crafted five processing steps (Fig. 2) to harmonize these scans. First, all PET images were converted into SUV units by normalizing the activity concentration to the dosage of 18F-FDG injected and the patient body weight after decay correction, and all CT images were converted into lung window. Second, spatial alignment of PET and CT images was performed using the affine registration in Elastix software (version 5.0.1), the quality of the alignment was visually inspected and the results were manually fine-tuned using 3D Slicer (version 4.11) to ensure accurate tumor region alignment. Third, the CT and PET images were normalized and resampled to an identical voxel resolution of 1×1×1 mm3. Fourthly, super-resolution reconstruction of resampled PET and CT images was performed using the Onekey platform (version 3.1, Beijing, China), its basic architecture is a generative adversarial network, which is a type of deep learning model comprising a generator network and a discriminator network. This study increased the spatial resolution of medical images fourfold while maintaining the original image size, transforming a voxel volume of 1 × 1 × 1 mm³ into a 1 × 1 × 0.25 mm³ volume, significantly improving image quality and spatial resolution. Finally, the images were discretized with a CT image bin width of 25 HU and a PET image bin width of 0.1 SUV.

**Tumor segmentation**

Tumor regions were selected as regions of interest (ROIs) on CT images and tumor boundaries were carefully identified, and combined with PET images when CT was difficult to distinguish. The outlined ROIs were then matched to PET images for further analysis. The tumor segmentation was independently performed by three imaging/nuclear medicine physicians who were blinded to the patients' clinical and pathological information. The segmentation results were comprehensively cross-examined by the three physicians, and the accuracy and consistency of the results were ensured through negotiation. This rigorous approach was taken to ensure the reliability and precision of our tumor region annotations. The SUVmax, SUVmean, MTV, and TLG of the segmented regions were calculated using the PET-IndiC, an extension of 3D slicer.


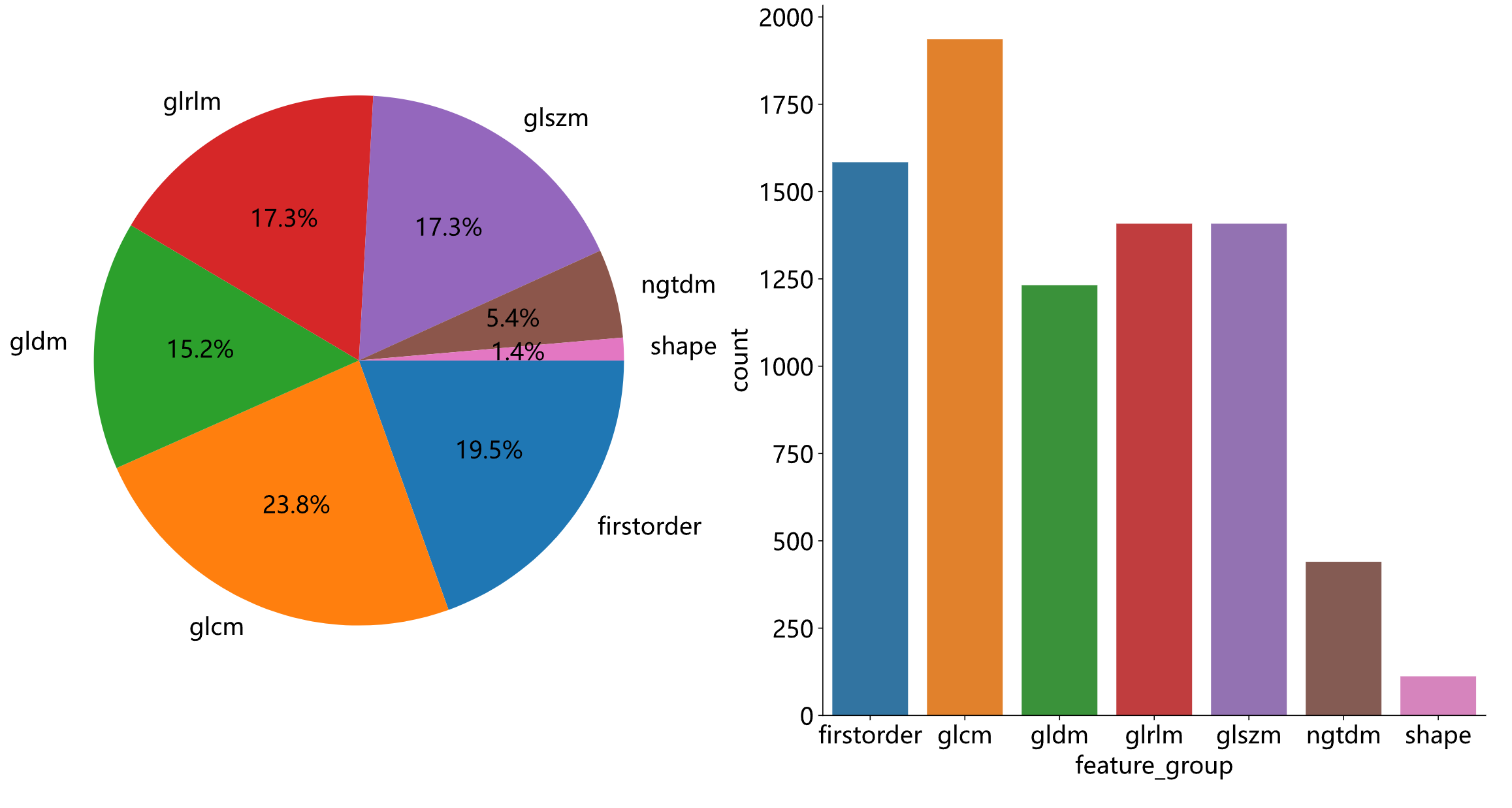


**Fig 1. Distribution of radiomic features of 18F-FDG PET/CT metabolic habitats**

glcm = gray level co-occurrence matrix, gldm = gray level dependence matrix, glrlm = gray level run length matrix, glszm = gray level size zone matrix, ngtdm = neighboring gray tone difference matrix.


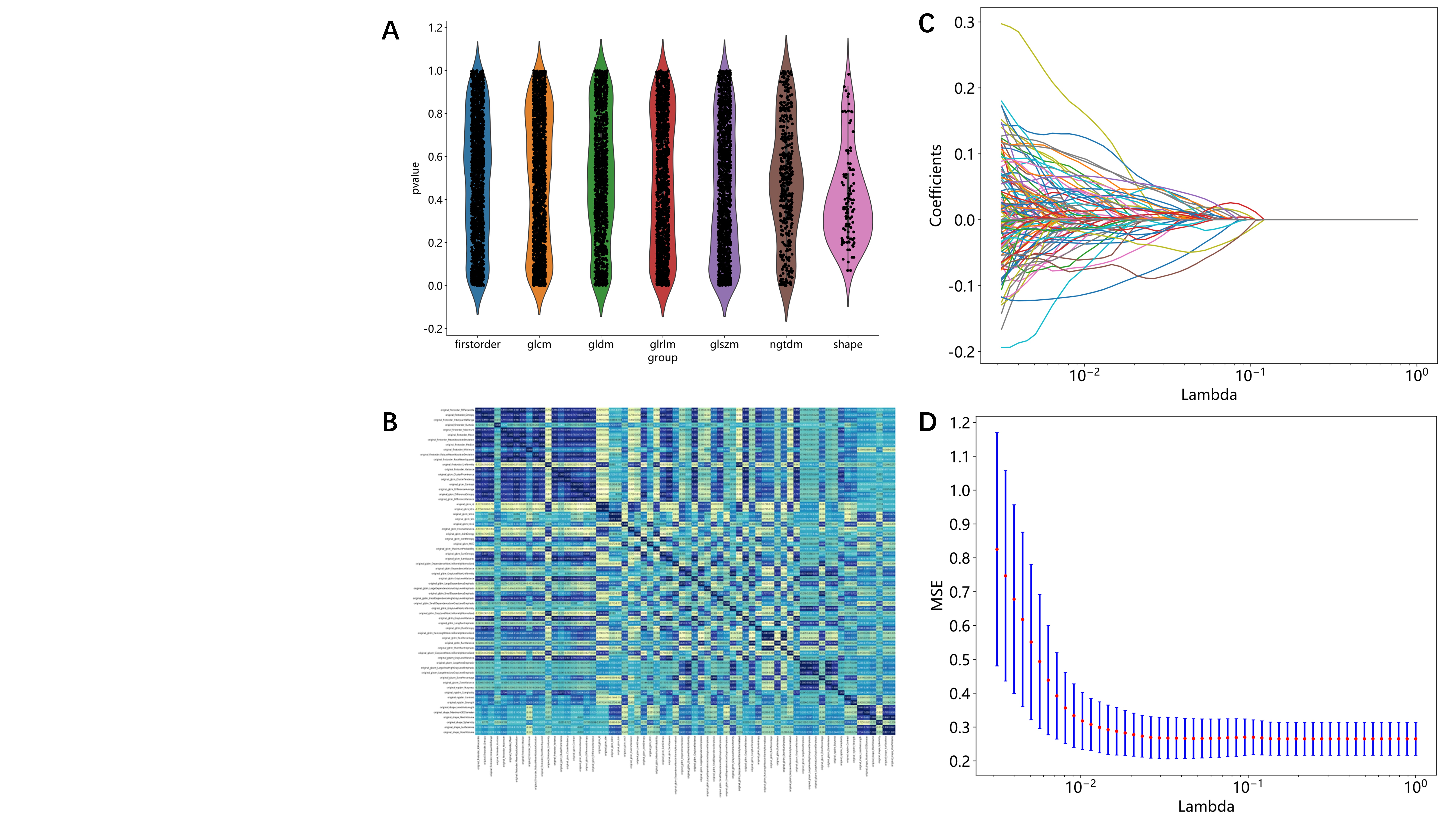


**Fig 2. Screening for** **radiomic features of ^18^F-FDG PET/CT metabolic habitats**

A. Violin plots from the two-sample Mann-Whitney *U*-test showed the relationship between radiomic features of metabolic habitats and PD-L1 expression. B. Spearman rank correlation coefficient was used to reduce redundancy. If the absolute value of the correlation coefficient between any two features was >0.9, only one was retained. C and D. Least Absolute Shrinkage and Selection Operators procedure (LASSO) with 10-fold cross-validation was used to select the most useful predictive features with non-zero coefficient.


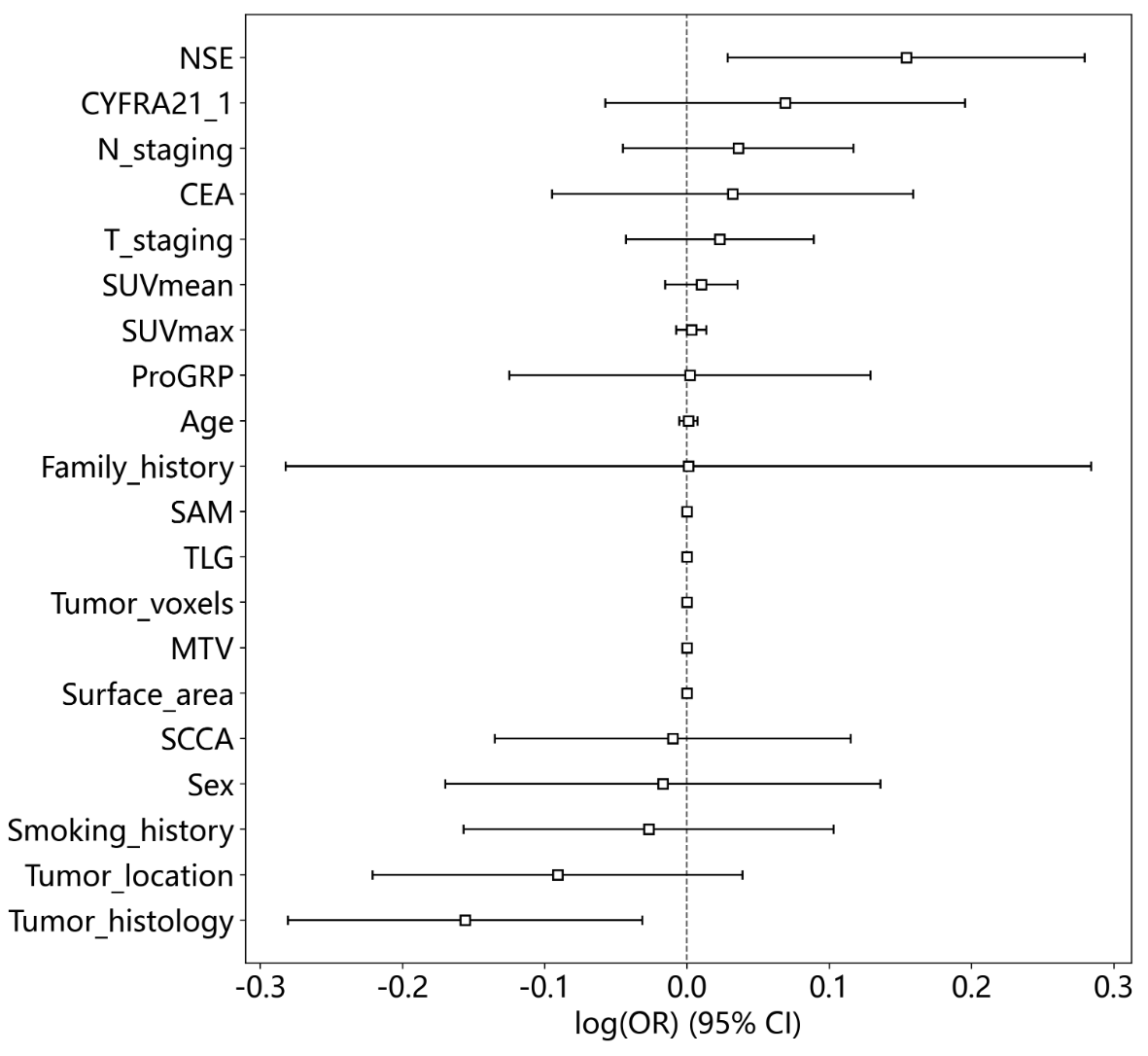


**Fig 3. Correlation between clinical characteristics and PD-L1 expression**

CEA = carcinoembryonic antigen, NSE=neuron specific enolase, CYFRA21-1 = soluble fragment of cytokeratin 19, ProGRP = pro-gastrin-releasing peptide, SCCA = squamous cell carcinoma antigen, SUV = standardized uptake value, MTV = metabolic tumor volume, TLG = total lesion glycolysis, SAM = Standardized Added Metabolic Activity.


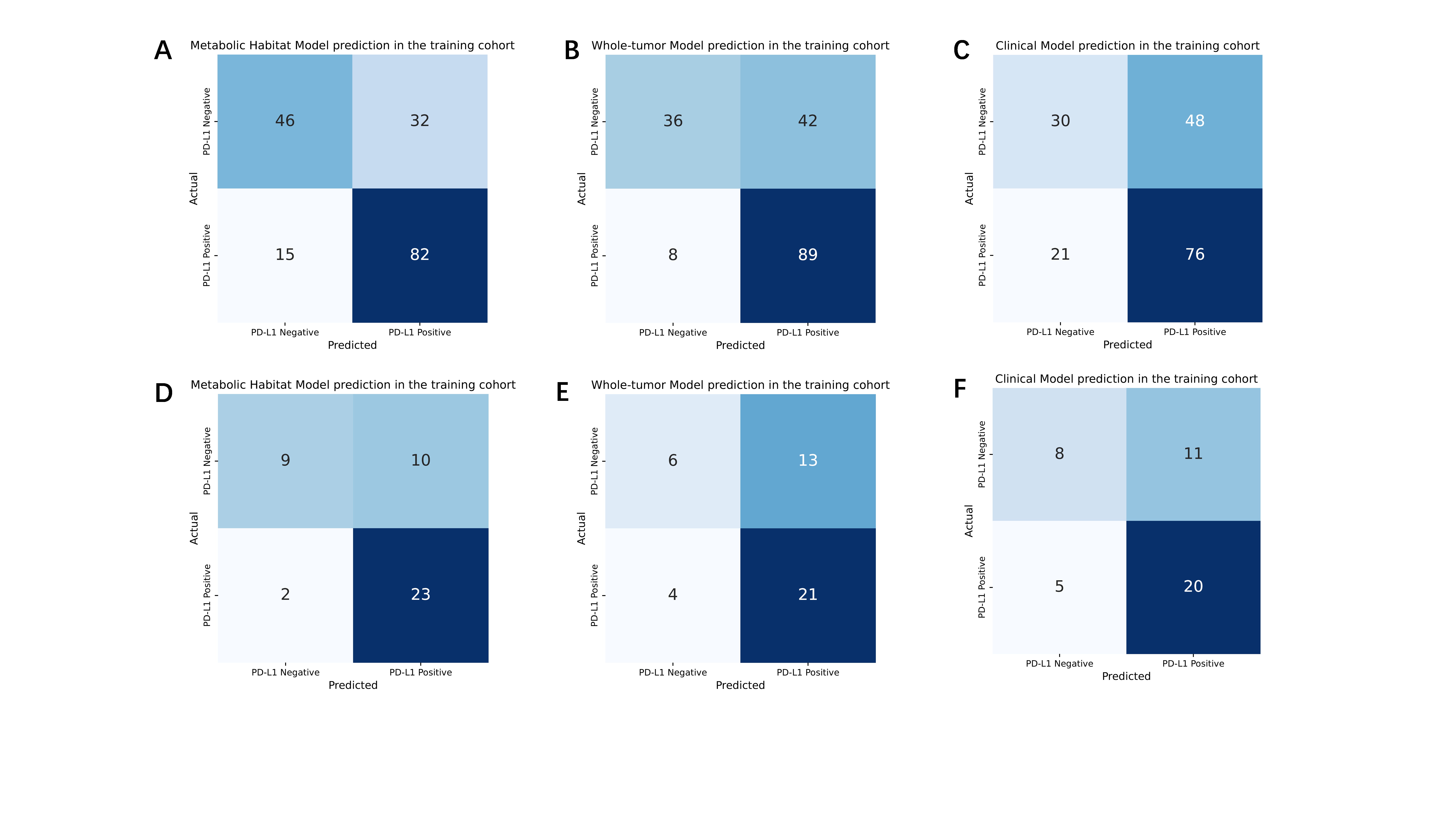


**Fig 4. Confusion matrix for metabolic habitat models, whole-tumor models, and clinical models.**

Confusion matrixes of the Extra Trees model to identify PD-L1 expression in the training cohort (A, B, C) and tesring cohort (D, E, F).


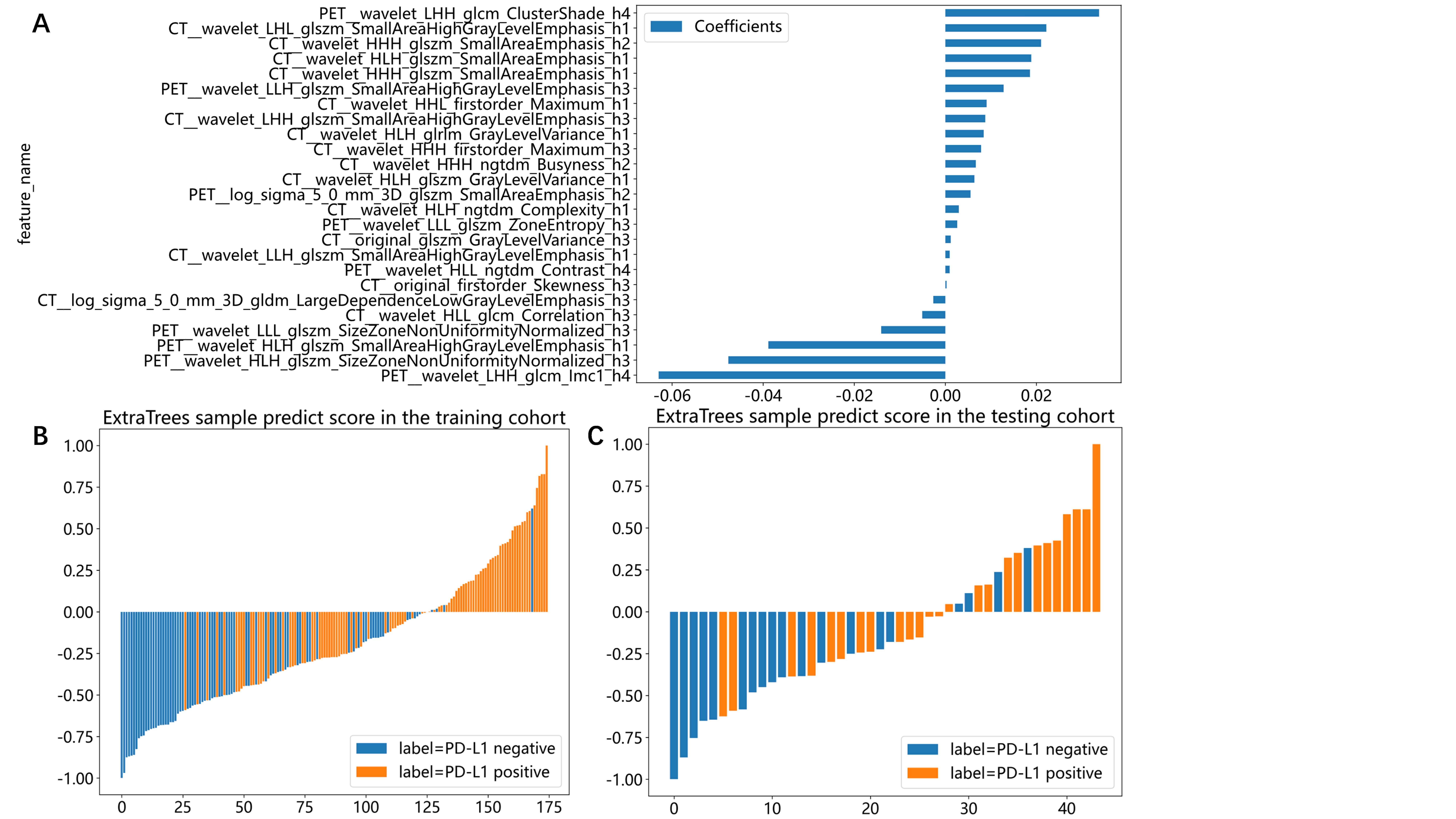


**Fig 5. Feature selection results and predicted waterfall plot.**

A. The final retained features after selection and their corresponding coefficients. B and C. Waterfall plot of prediction of PD-L1 expression to the proposed radiomic features for training cohort and testing cohort.


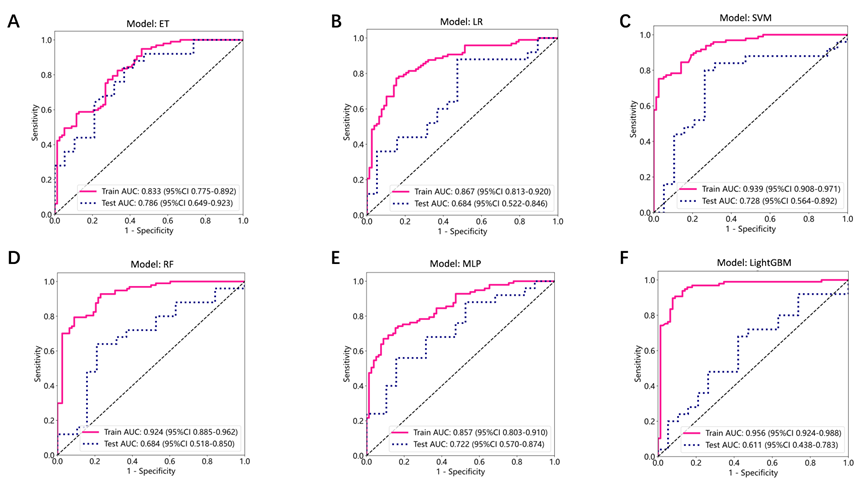


**Fig 6.** **Comparison of ROC curves among different machine learning models**

The ROC curve shows the predictive performance of models ET (A), LR (B), SVM (C), RF (D), MLP (E), and LightGBM (F) on PD-L1 expression in LA-NSCLC. Source data are provided in Supplementary Table 3.

ET = Extra Trees, LR = Logistic Regression, SVM = Support Vector Machine, RF = Random Forest, MLP = Multilayer Perceptron, LightGBM = Light Gradient Boosted Machine, LA-NSCLC = Locally advanced non-small cell lung cancer.


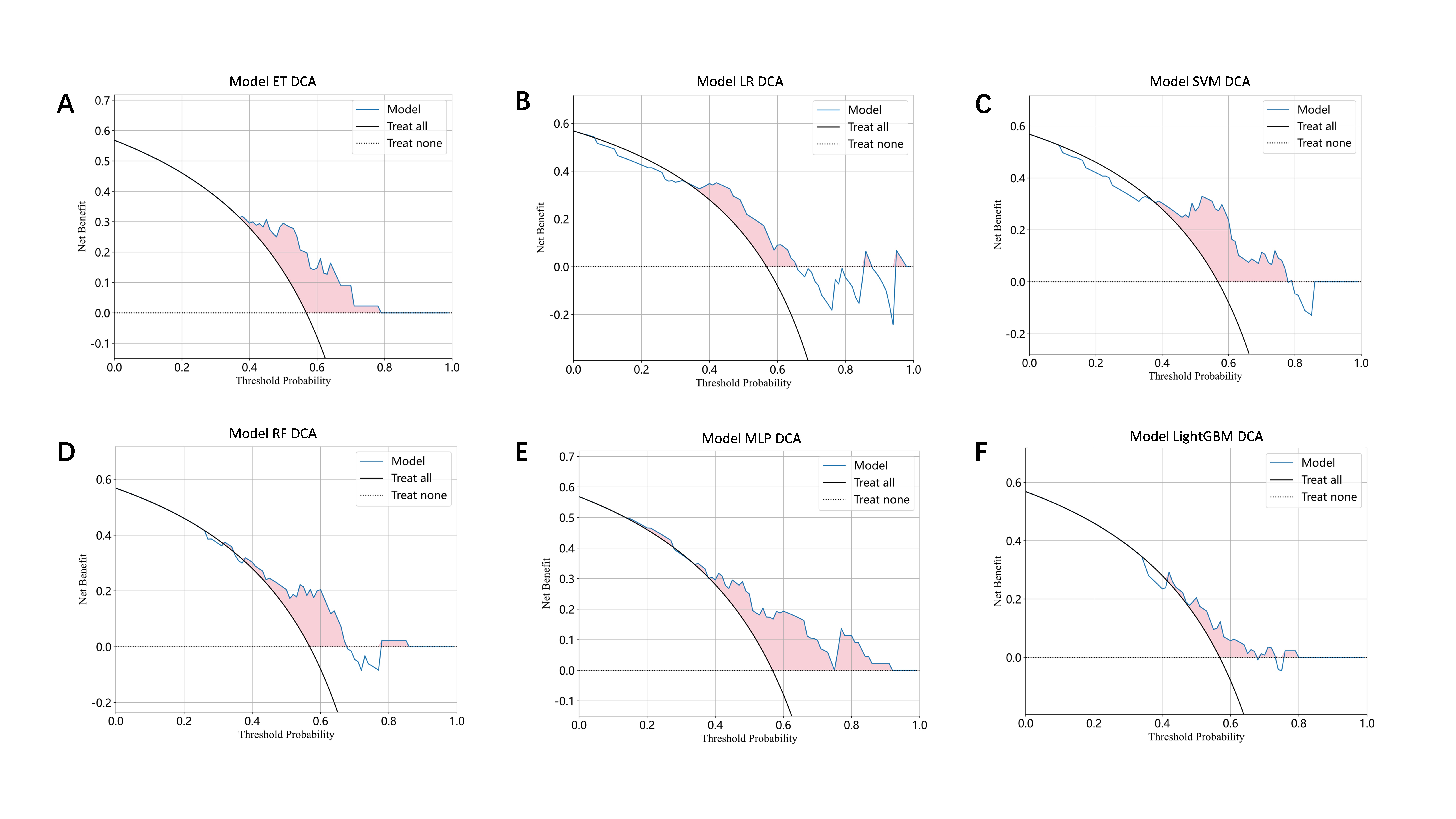


**Fig 7.** **Comparison of DCA curves among different machine learning models**

The DCA curve shows the overall net benefit of models ET (A), LR (B), SVM (C), RF (D), MLP (E), and LightGBM (F) on PD-L1 expression in LA-NSCLC. Source data are provided in Supplementary Table 3.

ET = Extra Trees, LR = Logistic Regression, SVM = Support Vector Machine, RF = Random Forest, MLP = Multilayer Perceptron, LightGBM = Light Gradient Boosted Machine, LA-NSCLC = Locally advanced non-small cell lung cancer.


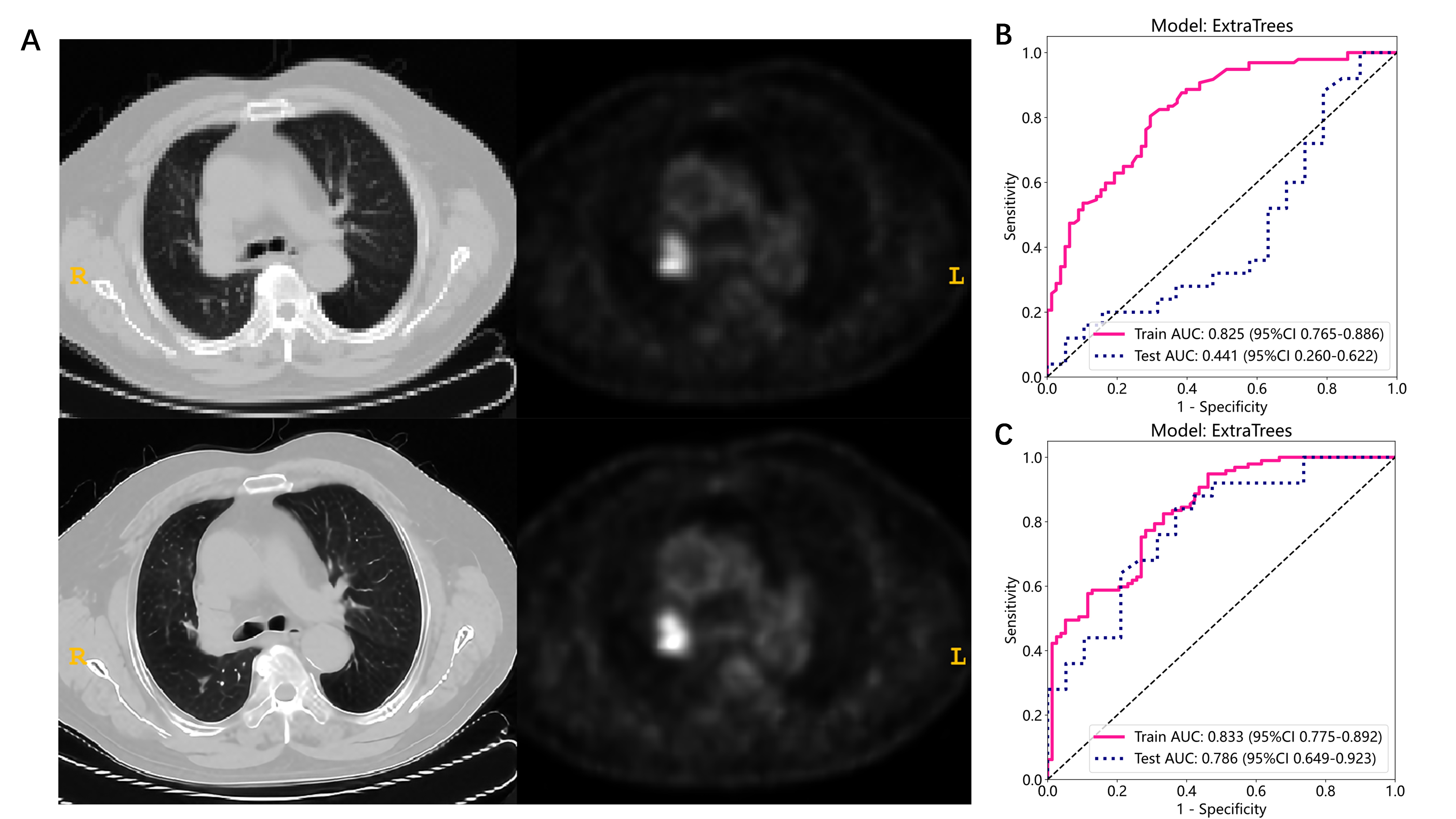


**Fig 8.** **Comparison between before and after super-resolution reconstruction**

A. Comparison of CT and PET images before and after super-resolution reconstruction. B and C. Comparison of ROC curves before and after super-resolution reconstruction based on Extra Trees model.

Table 1. Univariate and multivariate analysis of clinical characteristics

| Variable | Univariate analysis | | | Multivariate analysis | | |
| --- | --- | --- | --- | --- | --- | --- |
|  | Odds ratio | 95% CI | *p* value | Odds ratio | 95% CI | *p* value |
| Age | 1.013 | 0.985-1.042 | 0.355 |  |  |  |
| Sex | 0.784 | 0.415-1.482 | 0.454 |  |  |  |
| Smoking history | 1.142 | 0.659-1.979 | 0.637 |  |  |  |
| Family history | 0.923 | 0.300-2.842 | 0.889 |  |  |  |
| Tumor histology | 0.535 | 0.310-0.921 | 0.024 | 0.836 | 0.739-0.946 | 0.018 |
| Tumor location | 0.136 | 0.374-1.143 | 0.136 |  |  |  |
| T staging | 1.044 | 0.790-1.379 | 0.762 |  |  |  |
| N staging | 1.292 | 0.885-1.888 | 0.185 |  |  |  |
| Clinical staging | 1.233 | 0.844-1.801 | 0.279 |  |  |  |
| CEA | 1.271 | 0.739-2.184 | 0.386 |  |  |  |
| NSE | 1.735 | 1.003-3.001 | 0.049 | 1.194 | 1.053-1.353 | 0.02 |
| CYFRA-211 | 1.500 | 0.874-2.574 | 0.141 |  |  |  |
| ProGRP | 1.302 | 0.748-2.266 | 0.351 |  |  |  |
| SCCA | 1.081 | 0.634-1.843 | 0.775 |  |  |  |
| Tumor voxels | 1.000 | 1.000-1.000 | 0.347 |  |  |  |
| Surface area | 1.000 | 1.000-1.000 | 0.276 |  |  |  |
| SUVmax | 1.025 | 0.978-1.974 | 0.302 |  |  |  |
| SUVmean | 1.053 | 0.944-1.175 | 0.355 |  |  |  |
| MTV | 1.001 | 0.998-1.004 | 0.355 |  |  |  |
| TLG | 1.000 | 1.000-1.001 | 0.306 |  |  |  |
| SAM | 1.000 | 1.000-1.001 | 0.239 |  |  |  |

CEA = carcinoembryonic antigen, NSE=neuron specific enolase, CYFRA21-1 = soluble fragment of cytokeratin 19, ProGRP = pro-gastrin-releasing peptide, SCCA = squamous cell carcinoma antigen, SUV = standardized uptake value, MTV = metabolic tumor volume, TLG = total lesion glycolysis, SAM = Standardized Added Metabolic Activity.

**Table 2. The performance comparison of the metabolic habitat model, whole-tumor model, and clinical model.**

| Cohort | Model | Accuracy | AUC (95% CI) | Sensitivity | Specificity | PPV | NPV |
| --- | --- | --- | --- | --- | --- | --- | --- |
| train | Metabolic Habitat | 0.749 | 0.833 (0.775-0.892) | 0.814 | 0.667 | 0.752 | 0.743 |
| test | Metabolic Habitat | 0.727 | 0.786 (0.649-0.924) | 0.800 | 0.632 | 0.741 | 0.706 |
| train | Whole-tumor | 0.714 | 0.806 (0.743-0.869) | 0.649 | 0.795 | 0.797 | 0.646 |
| test | Whole-tumor | 0.636 | 0.639 (0.466-0.812) | 0.560 | 0.737 | 0.737 | 0.560 |
| train | Clinical | 0.571 | 0.621 (0.541-0.701) | 0.515 | 0.641 | 0.641 | 0.515 |
| test | Clinical | 0.568 | 0.581 (0.406-0.756) | 0.560 | 0.579 | 0.636 | 0.500 |

**Table 3. Comparison of performance between different machine learning models**

| Cohort | Model | Accuracy | AUC (95% CI) | Sensitivity | Specificity | PPV | NPV |
| --- | --- | --- | --- | --- | --- | --- | --- |
| train | ET | 0.749 | 0.833 (0.775-0.892) | 0.814 | 0.667 | 0.752 | 0.743 |
| test | ET | 0.727 | 0.786 (0.649-0.924) | 0.800 | 0.632 | 0.741 | 0.706 |
| train | LR | 0.800 | 0.867 (0.813-0.920) | 0.763 | 0.846 | 0.86 | 0.742 |
| test | LR | 0.705 | 0.684 (0.522-0.846) | 0.840 | 0.526 | 0.700 | 0.714 |
| train | SVM | 0.846 | 0.939 (0.908-0.971) | 0.742 | 0.974 | 0.973 | 0.752 |
| test | SVM | 0.750 | 0.728 (0.564-0.893) | 0.760 | 0.737 | 0.792 | 0.700 |
| train | RF | 0.840 | 0.924 (0.885-0.962) | 0.784 | 0.910 | 0.916 | 0.772 |
| test | RF | 0.682 | 0.684 (0.518-0.850) | 0.600 | 0.789 | 0.789 | 0.600 |
| train | MLP | 0.771 | 0.857 (0.803-0.910) | 0.660 | 0.910 | 0.901 | 0.683 |
| test | MLP | 0.659 | 0.722 (0.570-0.875) | 0.520 | 0.842 | 0.812 | 0.571 |
| train | LightGBM | 0.903 | 0.956 (0.924-0.988) | 0.887 | 0.923 | 0.935 | 0.867 |
| test | LightGBM | 0.614 | 0.611 (0.438-0.783) | 0.640 | 0.579 | 0.667 | 0.550 |

ET = Extra Trees, LR = Logistic Regression, SVM = Support Vector Machine, RF = Random Forest, MLP = Multilayer Perceptron, LightGBM = Light Gradient Boosted Machine, AUC= Area Under Curve, PPV= Positive Predictive Value, NPV= Negative Predictive Value.

**Table 4. Comparison of metabolic parameters in different habitats.**

| Cohort | Metabolic Parameters | Metabolic Habitat | | | | *P* value | | | | | |
| --- | --- | --- | --- | --- | --- | --- | --- | --- | --- | --- | --- |
|  |  | Habitat 1 | Habitat 2 | Habitat 3 | Habitat 4 | Habitat1 vs Habitat2 | Habitat1 vs Habitat3 | Habitat1 vs Habitat4 | Habitat2 vs Habitat3 | Habitat2 vs Habitat4 | Habitat3 vs Habitat4 |
| Train |  |  |  |  |  |  |  |  |  |  |  |
|  | Max | 13.86±0.45 | 6.84±0.18 | 6.93±0.18 | 11.35±0.42 | ＜0.001 | ＜0.001 | ＜0.001 | 0.737 | ＜0.001 | ＜0.001 |
|  | Mean | 9.23±0.27 | 4.29±0.11 | 4.76±0.12 | 8.26±0.25 | ＜0.001 | ＜0.001 | 0.059 | 0.041 | ＜0.001 | ＜0.001 |
|  | MTV | 29.41±3.44 | 8.82±0.94 | 29.35±3.02 | 4.26±0.82 | ＜0.001 | 0.985 | ＜0.001 | ＜0.001 | 0.185 | ＜0.001 |
|  | TLG | 305.81±38.83 | 43.04±5.66 | 146.91±16.04 | 44.42±9.30 | ＜0.001 | ＜0.001 | ＜0.001 | 0.001 | 0.965 | 0.001 |
| Test |  |  |  |  |  |  |  |  |  |  |  |
|  | Max | 13.39±0.84 | 6.88±0.40 | 6.89±0.40 | 11.43±0.85 | ＜0.001 | ＜0.001 | 0.007 | 0.961 | ＜0.001 | ＜0.001 |
|  | Mean | 9.21±0.56 | 4.33±0.27 | 4.65±0.27 | 8.45±0.53 | ＜0.001 | ＜0.001 | 0.395 | 0.521 | ＜0.001 | ＜0.001 |
|  | MTV | 27.86±7.94 | 10.22±2.51 | 23.39±4.94 | 4.45±1.36 | 0.012 | 0.520 | ＜0.001 | 0.059 | 0.409 | 0.007 |
|  | TLG | 293.46±82.53 | 51.17±14.09 | 132.34±31.93 | 49.10±15.62 | 0.005 | 0.013 | ＜0.001 | 0.210 | 0.020 | 0.201 |

SUV = standardized uptake value, MTV = metabolic tumor volume, TLG = total lesion glycolysis.
